# Genome sequencing as a generic diagnostic strategy for rare disease

**DOI:** 10.1186/s13073-024-01301-y

**Published:** 2024-02-14

**Authors:** Gaby Schobers, Ronny Derks, Amber den Ouden, Hilde Swinkels, Jeroen van Reeuwijk, Ermanno Bosgoed, Dorien Lugtenberg, Su Ming Sun, Jordi Corominas Galbany, Marjan Weiss, Marinus J. Blok, Richelle A. C. M. Olde Keizer, Tom Hofste, Debby Hellebrekers, Nicole de Leeuw, Alexander Stegmann, Erik-Jan Kamsteeg, Aimee D. C. Paulussen, Marjolijn J. L. Ligtenberg, Xiangqun Zheng Bradley, John Peden, Alejandra Gutierrez, Adam Pullen, Tom Payne, Christian Gilissen, Arthur van den Wijngaard, Han G. Brunner, Marcel Nelen, Helger G. Yntema, Lisenka E. L. M. Vissers

**Affiliations:** 1 Department of Human Genetics, Radboudumc, Nijmegen, Netherlands; 2 Research Institute for Medical Innovation, Radboudumc, Nijmegen, Netherlands; 3 Department of Clinical Genetics, Maastricht University Medical Center, Maastricht, Netherlands; 4 Illumina Inc., Cambridge, UK

**Keywords:** Rare disease, Genome sequencing, Impact modeling, Reducing workflow complexity, Genetic diagnostic laboratories, Germline variant detection

## Abstract

**Background:**

To diagnose the full spectrum of hereditary and congenital diseases, genetic laboratories use many different workflows, ranging from karyotyping to exome sequencing. A single generic high-throughput workflow would greatly increase efficiency. We assessed whether genome sequencing (GS) can replace these existing workflows aimed at germline genetic diagnosis for rare disease.

**Methods:**

We performed short-read GS (NovaSeq™6000; 150 bp paired-end reads, 37 × mean coverage) on 1000 cases with 1271 known clinically relevant variants, identified across different workflows, representative of our tertiary diagnostic centers. Variants were categorized into small variants (single nucleotide variants and indels < 50 bp), large variants (copy number variants and short tandem repeats) and other variants (structural variants and aneuploidies). Variant calling format files were queried per variant, from which workflow-specific true positive rates (TPRs) for detection were determined. A TPR of ≥ 98% was considered the threshold for transition to GS. A GS-first scenario was generated for our laboratory, using diagnostic efficacy and predicted false negative as primary outcome measures. As input, we modeled the diagnostic path for all 24,570 individuals referred in 2022, combining the clinical referral, the transition of the underlying workflow(s) to GS, and the variant type(s) to be detected.

**Results:**

Overall, 95% (1206/1271) of variants were detected. Detection rates differed per variant category: small variants in 96% (826/860), large variants in 93% (341/366), and other variants in 87% (39/45). TPRs varied between workflows (79–100%), with 7/10 being replaceable by GS. Models for our laboratory indicate that a GS-first strategy would be feasible for 84.9% of clinical referrals (750/883), translating to 71% of all individuals (17,444/24,570) receiving GS as their primary test. An estimated false negative rate of 0.3% could be expected.

**Conclusions:**

GS can capture clinically relevant germline variants in a ‘GS-first strategy’ for the majority of clinical indications in a genetics diagnostic lab.

**Supplementary Information:**

The online version contains supplementary material available at 10.1186/s13073-024-01301-y.

## Background

Although human genetic diseases are rare, they account for an important public health burden [1, 2]. Diagnostic approaches to detect the underlying genetic causes of these diseases require a broad spectrum of technologies, ranging from traditional approaches such as karyotyping, genomic microarrays, FISH, MLPA, and Sanger sequencing, to more advanced technologies, such as exome sequencing and transcriptomics. Each of these technologies is dedicated to detecting one or multiple variant types [3–6]. In clinical genomics, (de novo) single nucleotide and copy number variants (SNV/CNV) are the most commonly found aberrations [7–9], but to a lesser extent aneuploidy, expansions of short tandem repeats (STR), and (copy-neutral) structural variants (SV) also contribute to disease. To molecularly diagnose a rare disease, multiple workflows are often used, as a single disease can often be caused by multiple variant types [10–14]. Importantly, for diagnostic purposes, every technology needs to prove clinical, as well as analytical, validity [3, 15].

Genome sequencing (GS) promises comprehensive variant calling of all variant types from a single experiment, allowing for all types of molecular diagnoses [16, 17]. This (potentially) not only leads to an increased diagnostic yield but also provides a higher efficiency for genetic diagnostic laboratories that would no longer need to maintain multiple workflows to capture the various variant types. So far, however, widespread implementation of GS is lagging as the increase in diagnostic yield has been limited while incurring higher costs compared to routine workflows [18–20].

Several studies have performed direct comparisons between GS and one or a few techniques to explore concordance and utility [18–21], and GS has meanwhile been implemented for diagnosis and discovery in a few countries [22, 23]. A less explored scenario for effective implementation of GS as a routine diagnostic test is the impact of GS replacing all currently used diagnostic workflows. For instance, in our tertiary referral centers for genetic diagnostic testing at the Radboud University Medical Center (Radboudumc) and Maastricht University Medical Center + (MUMC +), approximately 25,000 individuals with a rare disease are tested annually, requiring > 10 molecular and cytogenetic workflows to capture all genetic variant types. Replacing these workflows with a single GS-based workflow would increase efficiency. To determine the feasibility of transitioning to a generic GS diagnostic workflow, we performed a benchmarking study using GS on 1000 individuals previously molecularly diagnosed with a rare genetic disease, representative of the myriad of genetic variant types identified across 10 different workflows and modeled the impact of a GS-first diagnostic strategy for rare disease in our centers.

## Methods

### Cohort selection

We retrospectively selected archival residual DNA material from a cohort (*n* = 1000) with known clinically relevant variants (*n* = 1271) from genome diagnostic laboratories of the Radboudumc in Nijmegen and the MUMC + in Maastricht. The cohort was selected from all positive reports in 2018, taking into account the distribution of molecular and cytogenetic workflows used in these departments for the primary diagnosis of germline variants underlying hereditary and congenital diseases using blood-derived DNA (based on the total number of requests and diagnostic yield), as well as to include a myriad of different genes, chromosomes and variant types (*n* = 979 cases, 1249 variants; Additional file 1: Table S1 and S2). The cohort was complemented with a few interesting cases for which DNA was extracted from another source than EDTA blood (*n* = 21, 22 variants; Additional file 1: Table S1 and S2). Of note, the cohort included 62 cases with diagnostic referrals that are under suspicion of harboring variants that are at risk to fail detection in a 30 × short-read genome. These cases had variants (*n* = 119 in genes or regions with a high level of sequence homology (*n* = 63), or possible mosaic variants (*n* = 56, range 2.4–54%), where the primary diagnostic referral was not always aimed at germline testing, but EDTA blood samples were available (Additional file 1: Table S2). Based on the selection criteria, the cohort is considered representative for our diagnostic centers.

### Genome sequencing

GS, using 150 bp paired-end short-reads, was performed as defined by the manufacturer (Illumina, San Diego, CA, USA). In brief, 1000 ng DNA was used for library preparation using the Illumina DNA PCR-free protocol and DNA was tagmented to an average insert size of 450 bp using bead-linked transposomes [24]. To allow equimolar pooling of samples, barcoded dual indexing was used after which the Illumina index correction strategy was applied (Additional file 1: Table S1). Sequencing was performed on an Illumina NovaSeq6000™ Instrument (24 samples on a S4 flowcell) to an anticipated genome-wide coverage of 30-fold minimal.

### Data analysis

Raw output was stored in Illumina’s BaseSpace Sequence Hub and data was analyzed using the Germline Pipeline of Illumina’s DRAGEN™ (Dynamic Read Analysis for GENomics) Bio-IT platform v3.7.5 [25, 26]. In short, after data is demultiplexed, mapped, and aligned (GRCh37), the DRAGEN Germline Pipeline provides a comprehensive analysis, including small variant (SNV and indels < 50 bp), ROH, CNV, and SV calling, as well as repeat expansion detection and genotyping through Illumina Expansion Hunter [27]. In addition, we used newly developed DRAGEN SMA [28] and CYP21A2 (DRAGEN v3.9) callers for those specific cases in which the genetic variants located in *SMN1/2* or *CYP21A2* (*n* = 19 cases, 34 variants).

### Variant detection strategy

Variant detection was divided into two phases. First, variant call format files (VCF) generated by the DRAGEN Germline Pipeline were assessed by automated (including clinical filters) or manual targeted queries using Illumina’s TruSight Software Suite v2.5 (TSS) to identify the variants of interest, resulting in a positive “ + ” (detected) or negative “ − ” (not detected) result. Variant detection was based on matching of chromosomal coordinates of small variants, or reciprocal overlap of genomic event intervals for large variants (CNV, ROH, STR). Other variants (structural variants and aneuploidies) were only investigated manually using VCFs and the Integrative Genomics Viewer (IGV) genome browser [29] in TSS, as TSS did not support automated features (clinical filters) at the time of analysis. Second, variants that failed detection were further assessed to determine why they were absent from the VCFs.

### Sensitivity analysis

Sensitivity analysis was performed in two ways. First, we assessed the overall sensitivity of GS by calculating the true positive rate (TPR) for each workflow, defined as the number of true positive variants (TP) divided by the total number of variants (*n* = 1271 in 1000 cases) including the false negatives (FN; TPR = TP/(TP + FN). Second, we repeated the analysis after exclusion of the cases (*n* = 62) with variants (*n* = 123) which were a priori known to fail detection in a 30 × short-read genome to better approximate the TPR.

### Impact analysis

We modeled a scenario of the overall impact of GS implementation as a generic workflow. Hereto we performed three in silico analyses.

First, we determined the sequence depth at genomic positions that are known to harbor (likely) pathogenic variation. Sequence depth was calculated from 35 randomly selected genomes. The median coverages were subsequently intersected with genomic positions (coordinates) of all known pathogenic variants reported in the repository of the Dutch Association of Clinical Laboratory Geneticists [30, 31] and ClinVar [32]. In addition, we determined the median coverages for all coding positions of genes with well-established rare disease associations [33]. Under the assumption that sequence coverage is one of the main determinants for being able to reliably call a variant, we next calculated the fraction of variants with sufficient coverage. Minimal threshold for presumed detection of a variant was set at tenfold coverage at the respective genomic coordinate. Assuming a binomial distribution with probability 0.5 of sequencing the variant allele at a heterozygous position, at least 10 reads are required to obtain a 99% probability that at least two reads contain the variant allele [34].

Secondly, we extrapolated and modeled the obtained workflow-based TPRs and GS variant detection limitations from our experimental data to a real-life scenario of our genetic diagnostic laboratories. In line with guidelines for assuring the quality of diagnostic next-generation sequencing [35, 36], we used a TPR of ≥ 98% as threshold for replacing workflows by GS. As input for our model, we used anonymized data of all 41,691 individuals tested in our genetic diagnostic laboratories in 2022 (Additional file 2: Fig. S1). For each diagnostic referral (*n* = 54,680), we evaluated the reason for referral and eligibility for inclusion in our model. A total of 24,166 referrals were excluded, as these either represented cascade screening (*n* = 7854) or were not within the current scope of replacing by GS (*n* = 16,312), such as for instance non-DNA based and/or biochemical assays (Additional file 2: Fig. S1). For the remaining 30,514 referrals for testing, performed in 24,570 individuals, we determined the experiments and workflows used to address the diagnostic referral as input for the model. Combining the workflow and variant type detected per clinical indication, we modeled the impact of substituting eligible experiments (clinical indications) for GS in the diagnostic trajectory of these individuals. Of note, for individuals with multiple referrals that could be replaced by GS, a maximum of one GS was considered, with subsequent diagnostic referrals involving reanalysis of existing data.

Finally, to determine the impact of the GS-first strategy on overall diagnostic yield, the outcome per individual was projected under the following assumptions:

- Negative diagnostic results remained negative, regardless of the underlying workflow, thus also *not* considering a possible added diagnostic value of GS.
- For individuals whose diagnostic track would not include GS, or where GS was supplemented with an additional non-GS transferable clinical referral, the original diagnostic outcome was maintained.
- For individuals with a conclusive ((highly) likely pathogenic variant), or possible (variant of unknown significance) diagnosis, the GS diagnostic outcome was offset with the TPRs per workflow. Of note, for individuals with multiple diagnostic referrals, it was first determined which experimental workflow led to the initial possible/conclusive diagnosis.

We subsequently determined the number of individuals negatively impacted by the GS-first strategy as proxy for false negatives [FN]. The false negative rate (FNR) was determined by FNR = [FN]/[FN] + [TP], in which [TP] was defined as the original diagnostic yield in the cohort of 24,570 individuals minus the [FN].

## Results

### Genome diagnostics and cohort demographics

This local 1000 genome project included archival DNA samples of 505 males and 495 females who were genetically tested in our laboratories using 10 different workflows (Additional file 1: Table S1; Additional file 2: Fig. S2). For 378 individuals, this included analysis of specific variants, a single gene, or a few genes, whereas in 617 individuals, extensive gene panels or other genome-wide analyses were used. For the remaining five individuals, a combination of both approaches was employed (Additional file 2: Fig. S2). A total of 1271 diagnostically relevant variants were reported (Additional file 1: Table S2; Additional file 2: Fig. S2). All variants were called complying to specifications of DRAGEN variant calling, grouping them in three categories: a category for small variants (*n* = 860), including SNVs and indels up to 50 bp in size, a second one for large variants (*n* = 366), i.e., CNVs and STRs, leaving a third category for all other variants (*n* = 45), involving SVs and chromosome anomalies (CA) (Additional file 1: Table S2; Additional file 2: Fig. S2). For our 1000 genomes, we reached an average sequencing depth of 37 × (Additional file 2: Fig. S3).

### GS technical validation and feasibility assessment of replacing workflows by GS

**Fig. 1:**
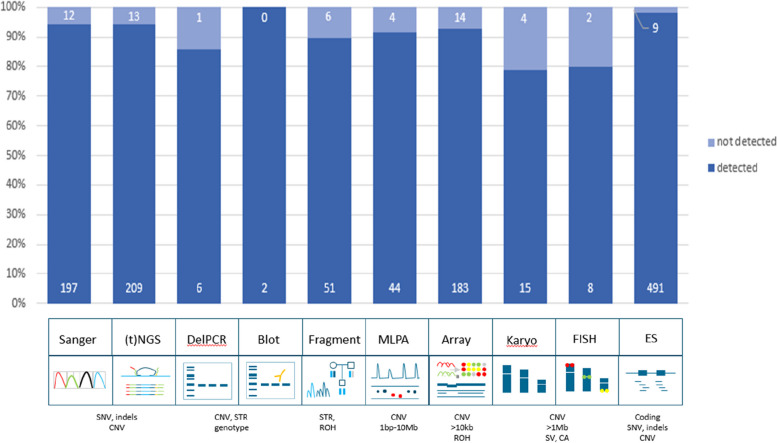
Technical validation of 1271 variants. Schematic representation of detection rates of previously identified pathogenic variants across multiple different workflows. In total, 94.9% (1206/1271) of all variants were detected in GS data. The distribution of variants across the ten workflows shows a detection rate ranging between 79 and 100%. Abbreviations: targeted next-generation sequencing ((t)NGS), deletion polymerase chain reaction (DelPCR), multiplex ligation-dependant probe amplification (MLPA), fluorescence in situ hybridization (FISH), exome sequencing (ES), single nucleotide variants (SNV), copy number variants (CNV), short tandem repeat expansions (STRs), region of homozygosity (ROH), structural variants (SV), chromosome anomalies (CA)

In total, 94.9% (1206/1271) of all variants were detected with GS (Fig. 1; Additional file 1: Table S2). Small variants were detected in 96.1% (826/860), large variants (123 bp–72.8 Mb) in 93.2% (341/366), and other variants in 86.7% (39/45) (Additional file 2: Fig. S4 and S5). Subdividing the cohort by the variants we expected to readily identify (*n* = 1152) and those that we would not (*n* = 119), indeed confirmed the prior knowledge of the technical challenges in detecting mosaic variants and variants located in homologous regions or genes with short-read 30 × GS: 1138 of 1152 variants (98.8%) were detected as expected, whereas only 68/119 (57.1%) of challenging variants were identified (Fisher’s exact test *p* < 0.001; Additional file 1: Table S2). The variants that remained undetected after manual curation (*n* = 65), could be categorized into four categories: mosaic variants (*n* = 27, including somatic and mitochondrial variants), homologous regions (*n* = 25, e.g., variants in *STRC* or the Opsin gene family), short tandem repeats/repetitive sequence (*n* = 10, such as *FMR1* and Robertsonian translocations), and other variants (*n* = 3). Of note, the detection limit of small mosaic variants was 13%.

We next reconstituted the 1271 variants to their original workflows to determine the overall performance of detection of different variant types per workflow, which ranged from 79% for karyotyping to 100% for Southern blots (Fig. 1). Subsequent analysis of the TPR per workflow revealed that all workflows, except repeat length analysis, karyotyping, and FISH, were determined to have a TPR > 98% (Additional file 2: Table S3).

### In silico extrapolation of detection rates to 58,393 variants and 4266 disease genes

Assessing the available coverage data of 794 detected SNVs in our cohort showed that 99.1% had a ≥ 10 × coverage (Additional file 1: Table S2; Additional file 2: Fig. S6). We next leveraged the observations onto a larger in silico data set of variants. Hereto, we obtained 58,393 genomic coordinates from variants known in the VKGL and/or ClinVar databases to cause autosomal dominant/recessive disease (Additional file 1: Table S4) and determined the sequence coverage for those positions across 35 genomes. For 99.5% of variants, the minimal coverage across 35 genomes was ≥ 10 × (Additional file 2: Fig. S6). Generation of similar coverage statistics for all coding bases of 4,266 disease-associated genes showed that the average coverage was 45 × (Additional file 1: Table S5; Additional file 2: Fig. S5), with 88.1% of genes (3759/4266) having a coverage of ≥ 10 × for all protein-coding bases (Additional file 2: Fig. S6).

### Modeling the impact of GS implementation in clinical practice

We next set out to model the impact of GS implementation on everyday practice in our clinical centers, from both the clinical point of view, as well as from the laboratory point of view. In addition, we determined the impact on overall diagnostic yield obtained from a GS-first perspective.

**Fig. 2:**
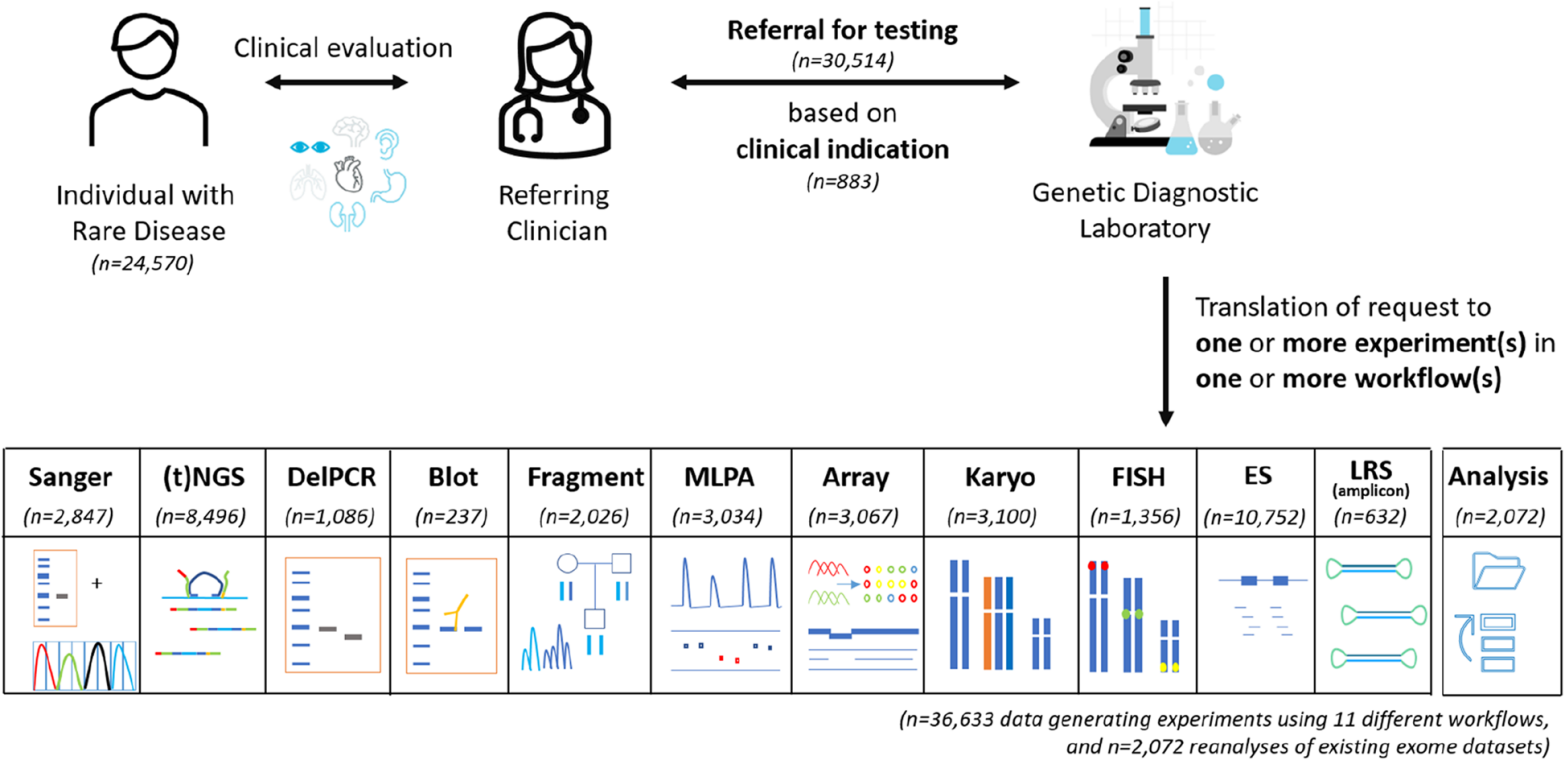
Diagnostic referrals for genetic testing in 2022. In total, 24,570 individuals were referred, together requiring 36,633 data-generating experiments (in 23,604 individuals) in 11 different workflows, and 2072 reanalyses of existing (exome) datasets (in 966 individuals). Abbreviations: targeted next-generation sequencing ((t)NGS), deletion polymerase chain reaction (DelPCR), multiplex ligation-dependant probe amplification (MLPA), fluorescence in situ hybridization (FISH), exome sequencing (ES), long-read sequencing (LRS)

In 2022, our tertiary genetic diagnostic laboratory received 30,514 diagnostic referrals to identify the primary germline DNA defect in 24,570 individuals with rare disease (Fig. 2; Additional file 2: Fig. S7). In total, 883 different reasons for referral were observed, with the top 10 ranking clinical indications being responsible for 21% of all referrals. On average, per individual 1.24 referrals were noted, and 82% of individuals were referred only once (Additional file 2: Fig. S7). Of note, for 966 individuals, the diagnostic referral (*n* = 2072) consisted of reanalysis of existing exome data and did not require the generation of novel experimental data. For the other 28,442 referrals, 36,633 wet lab experiments were performed using 11 different workflows (Fig. 2).

**Fig. 3:**
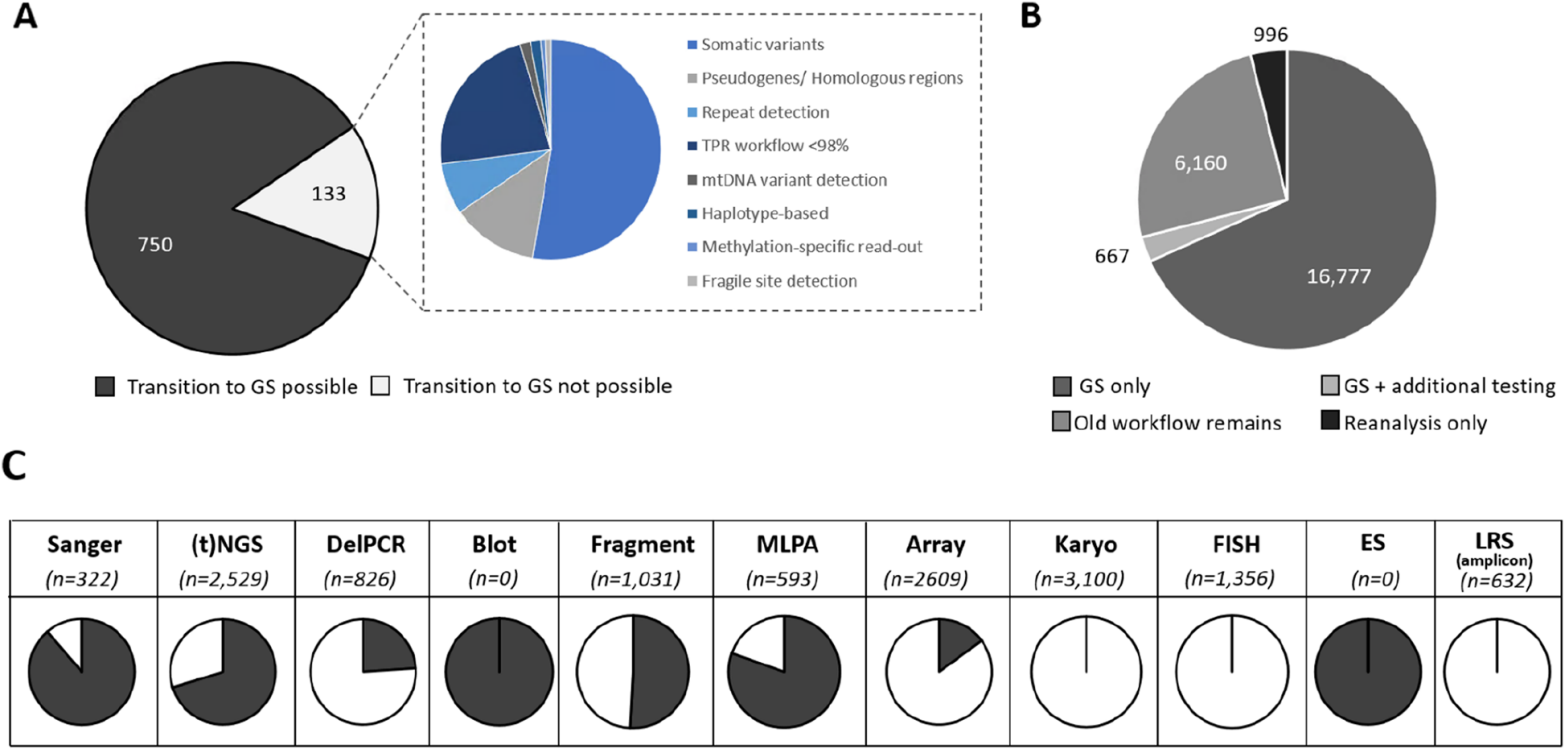
Assessing the impact of a GS-first transition. **A** From 833 different clinical reasons for referral in 2022, 750 can be transitioned to GS. **B** This transition would result in 16,777 individuals receiving GS as the only workflow. For 667 (3%), the GS should be supplemented by an additional test, whereas for the remaining 7126 (29%) GS would not be suited, either because for them the clinical indications included experiments not transferable to GS (*n* = 6160; 25%), or because the referral did not require data generation (*n* = 966; 4%).** C** The use of GS as a primary test has a significant impact on reducing the experimental workload in the original workflows. Proportions of the transferable number of tests per workflow are indicated in black. Abbreviations: targeted next-generation sequencing ((t)NGS), deletion polymerase chain reaction (DelPCR), multiplex ligation-dependant probe amplification (MLPA), fluorescence in situ hybridization (FISH), exome sequencing (ES), long-read sequencing (LRS)

From a clinical point of view, 750 of 883 (85%) clinical reasons for referral could be addressed via GS (Fig. 3). The remaining 133 could not be performed via GS for various reasons, of which somatic variant detection (53%) and detection of variants in homologous regions (13%) are the most prominent (Fig. 3). From a laboratory point of view, this GS-first strategy would not only fully replace the exome workflow and all Southern blots but would also considerably reduce the use of other workflows, such as Sanger sequencing (by 89%), MLPA (by 80%) and targeted NGS approaches (by 70%; Fig. 3). Importantly, applying these observations to the diagnostic trajectory of all individuals shows that GS can be used as first-tier test for 16,777 (68%; Fig. 3) of individuals.

Finally, we modeled the impact on the overall diagnostic yield. In 2022, a conclusive molecular diagnosis was obtained in 2652 of 24,570 individuals (10.79%), and for another 3597 (14.64%) a possible diagnosis was identified. Extrapolation of TPRs for individuals whose diagnostic trajectory would include GS, resulted in an anticipated conclusive diagnosis in 2643 individuals (10.76%) and a possible diagnosis in 3589 (14.61%; Additional file 2: Fig. S8). Collectively, a generic GS-first strategy would thus possibly negatively impact the diagnostic outcome for 17 (0.07%) individuals (FN = 17), translating to a possible false negative diagnostic rate of 0.3%.

## Discussion

Over the last decade, the use of GS as a routine diagnostic test has been debated in the context of a higher potential diagnostic yield by interpreting non-coding DNA variants, as well as the potential to diagnose individuals with rare disease more efficiently, as GS allows the identification of virtually all genetic variants in a single experiment. Widespread diagnostic implementation has however been hampered by the costs involved with GS, given that the anticipated higher diagnostic yield has so far not materialized. An increased diagnostic yield is however still expected for unexplained rare genetic disease, especially when looking beyond SNV and CNV detection in the exome only. To ultimately benefit from the advantages of GS, costs need to be reduced for a generic genetic diagnostic laboratory. In this study, we focused on the potential for GS as a generic diagnostic rare disease test, replacing the full spectrum of workflows available in a genetic diagnostic laboratory. With our cohort of 1000 genomes, representative of 10 different workflows and a multitude of genetic variant types, we found that GS detected > 95% of all pathogenic variants, albeit with variable efficacy across variant types and workflows. We also modeled the impact of a transition to a generic GS workflow for our diagnostic laboratories and concluded that for 68% of individuals diagnostically referred to our departments a generic GS workflow would be possible.

In our series of 1000 samples, we noted differences in the detection of different variant types; 96.1% of small variants (< 50 bp) were detected, whereas only 93.3% of large variants, and 86.7% of other variants were recovered from GS. Interestingly, one of the arguments generally used as benefit from GS is its ability to better detect structural variation compared to ES [37]. Conceptually, this is true from having a more uniform coverage across the genome [38]. In addition, we, and others, have previously shown that additional diagnoses obtained via GS compared to routine care, not only are often SVs, but also that the resolution of SV complexity identified, often (far) exceeds that of other technologies [20, 39]. However, our data now show that the capture of SNVs/indels from GS is more complete than of SVs (Fisher’s exact, *p* = 0.006). Irrespectively, it must be noted that the overall number of SVs evaluated was limited due to our inclusion criteria which required DNA from EDTA blood. Further retrospective analysis of pathogenic SVs might provide additional insights. Also, given the assumption and prior work showing that GS excels in SV detection, one might also speculate on GS now uncovering variants where the initial gold standard technology might have been wrong.

Any technology comes with technical limitations. Here, we did not identify any unexpected limitations in variant calling for the 5% (65/1271) of undetected variants, besides the already known limitations of variant calling in short-read GS data. For instance, it is known that mapping short reads in homologous regions is difficult, and also, short-read GS at ~ 30 × will pose difficulties in detecting mosaic variants. Solutions to recover these variants from short read data are, however, possible: for mosaic variants, increasing GS sequence depth may be able to recover all clinically relevant variation, while bioinformatic callers might help in the successful retrieval of (likely) pathogenic variants in complex homologous genomic regions. Currently, such dedicated callers already exist, e.g., we successfully used callers for the SMA [28] and *CYP21A2* loci in our analyses, and also, other tools calling variation in paralogous regions have been developed [40]. On a positive note, we already recovered 68 of 119 variants that we a priori expected to be beyond the technical limitations of 30 × GS, without further optimization. These included variants located in highly homologous regions (*STRC* and *OTOA*), as well as variants present in mosaic state (> 14%), for which in the SOC trajectory dedicated tests are deployed that are designed to detect specific variants of interest.

Diagnostic efficacy can be enhanced by reducing the complexity of sample handling and the number of workflows. In our laboratory set-up, one clinical referral is often translated into experiments in multiple workflows; for example, to molecularly diagnose CHARGE syndrome, caused by *CHD7* haploinsufficiency, both Sanger sequencing and MLPA analysis are needed to allow the detection of SNV/indels as well as of (partial) gene deletions. The introduction of a generic GS workflow would allow for calling both SNV/indels, CNVs, and other SVs affecting *CHD7* from a single experiment. For other disorders, for instance, those caused by the expansion of short tandem repeats, it might be more challenging, as short-read sequencing technologies may be unable to capture the full length of the extension. However, our data shows that although for some repeats the exact length cannot be obtained, a generic GS workflow is able to identify those individuals with repeat lengths outside of the normal range. This result can be followed with dedicated tests to determine the size of the repeat. From an efficacy point of view, one may argue that a second workflow is still required. While this is a valid point, in a generic GS workflow, the subsequent use of a second workflow is much more efficient, as it will only be used for those individuals with a high a priori chance of a positive outcome (given their abnormal GS results).

Whether or not it is efficient for laboratories to make a transition towards a generic GS workflow may depend on lab-specific factors, including size of the lab, number of workflows in use, and type of diagnostic referrals received. From our series of 1000 genomes tested, we showed that ES can technically be replaced by GS (TPR > 98%), in line with previous reports on comparing diagnostic outcomes of ES and GS [18–20]. Hence, diagnostic laboratories, whose expertise is to only perform ES, could easily move towards GS with the benefit of a faster workflow as enrichment is no longer needed [24]. Yet, for laboratories specialized in the use of karyotyping (TPR < 98%) for the detection of somatic copy number changes, routine 30 × GS might not be sufficient. Additionally, we showed that there are some genes with lower coverage (for part) of all protein-coding bases, which warrants caution when these genes are of specific interest related to a specific clinical differential diagnosis. The results of our study should therefore be carefully examined and extrapolated to local infrastructure and clinical expertise. Of note, a site-specific (early) health economic impact analysis is also recommended prior to large-scale implementation, in which evaluation of cost-effectiveness is key. These studies are mostly performed in the context of proving that an early diagnosis also has a beneficial impact on overall health care cost expenditure [21, 41–43]. In light of implementing a generic GS workflow, a complementary micro-costing study could be of relevance [44, 45]. Such studies would allow to weigh possible cost-reductions from phasing out workflows and changes in workforce against potential increase of per-sample sequencing costs, as well as the costs associated with (ease of) clinical data interpretation.

Here, we report on our laboratories, which together maintain > 10 workflows, representative for most core technologies used in genetic testing [16], and enabling detection of all variant types. The scenario models for our centers showed that 750/883 (85%) diagnostic referrals can be completed using GS, which would result in 68% of all individuals referred to our diagnostic laboratory making use of a single workflow and a single experiment, and 3% needing additional testing, suggesting that for 71% of individuals (*n* = 17,444) a GS-first strategy would be beneficial. Whereas our analysis did not include a full micro-costing study, a generic GS-first workflow for such volume of samples might become within reach, especially with prices announced for germline GS in the range of 100 to 200 dollars per genome [46–48]. For the 15% of clinical indications not transferable to GS (responsible for 29% of individuals referred), we noted trends, such that most of these required somatic structural variant detection, currently assayed via karyotyping, FISH and/or arrays, or variants were located in complex regions of the genome, currently assessed by amplicon-based long-read sequencing strategies [49]. These technical challenges can — in part — be overcome by sequencing at higher depth (e.g., ~ 100 × or even ~ 350 ×) to allow better somatic SNV/indel detection [50]. Yet, it could also be considered to maintain workflows for dedicated diagnostic referrals. Alternatively, technological innovations specifically targeting these more challenging variant types and regions would constitute a worthwhile investment. For somatic variant detection via karyotyping, FISH and/or arrays, optical genome mapping [51, 52] could replace these workflows as a second major generic assay, available in parallel to GS, but used for mutually exclusive clinical referrals. Similarly, a more generic use of long read genomes [53, 54] may provide a costs-effective strategy for diagnostic referrals involving variants in complex regions in the genome, or where variant size exceeds those detectable from short reads (such as for repeat expansions). For either technical solution, a careful evaluation of the required coverage, as well as the impact on the false negative rate when compared to the old technique [38, 55–58].

The implementation of a novel technology requires careful balancing of the pros and cons. For GS, our study has highlighted advantages related to laboratory efficiency, but also showed that not all previously detected (likely) pathogenic germline variants were also identifiable from GS. Hence, if a generic GS workflow were to be used, it is to be expected that some individuals who would receive a conclusive diagnosis with the old diagnostic test strategy, would no longer do so with the implementation of a generic GS. In our objective quantification of the false negative rate from GS, using all diagnoses obtained by the current diagnostic strategy as the gold standard, we modeled that the transition to a generic GS in our laboratory might result in an additional diagnostic false negative rate of 0.3%. Whereas this is undesirable for the individual patient, previous experience has shown that there may be trade-offs. For instance, with the introduction of genomic microarrays at the expense of karyotyping, no longer detecting apparently balanced chromosomal rearrangements had to be accepted. Further, with the introduction of ES as replacement for Sanger sequencing for genetically and clinically heterogeneous disorders, one lost sensitivity at base pair level while gaining in mutation target size. Both innovations changed diagnostic testing, because despite losing out on a few positive diagnoses, they still improved the overall diagnostic yield [59, 60]. So far, the overall diagnostic advantage of GS is still limited. Disease-specific evaluations of diagnostic yield of GS have, however, reported on an increase in diagnostic yield, ranging from 1.3% for neurodevelopmental disorders [20] to 17% for congenital limb malformations [17]. Additionally, it has been reported that cytogenetically found apparently balanced chromosomal rearrangements appear to be genomic imbalances in ~ 1/3 of patients with de novo translocations and inversions [61, 62], and that ~ 2/3 of balanced chromosomal abnormalities are involved in pathogenic mechanisms [63]. With growing experience in detecting and interpreting structural variants in GS data, we also expect to identify more inversions, translocations, and other structural variants as underlying causes of human genetic disease. The use of GS over current workflows would provide an added value for which individuals with rare disease would immediately benefit, thus potentially compensating for the 0.3% diagnostic loss from introducing a generic GS workflow.

Finally, our study is designed as technical benchmarking, which did not include an evaluation of variant prioritization. We and others have, however, recently shown in prospective parallel and randomized GS studies that similar variant types and diagnostic yield are obtained when comparing GS to current (non-GS) standard-of-care diagnostic workflows [18, 20]. In light of this, it is also worthwhile to underscore that even though analytically a full genome sequence is provided, a targeted interpretation of variants, in line with the clinical request, could still be pursued. For instance, initially, variants in single genes can be prioritized using in silico enrichment strategies when the GS is performed instead of a Sanger test, or, alternatively, only CNVs can be visualized when otherwise an array would have been analyzed. If negative, a more agnostic approach for the interpretation of genetic variation can performed, where the existing and available GS data provide a valuable resource for efficient reanalysis and reinterpretation strategies. We noted that 6.8% of our referrals (*n* = 2072) already involved reanalysis of existing exome data. With increasing knowledge on the role of (rare) non-coding variants in relation to disease and improvement in the bioinformatic detection of variants in complex regions of the genome from short reads, it can be expected that the availability of GS provides more flexibility in adapting reanalysis strategies towards these loci and variant types in the near future.

## Conclusions

In summary, our benchmarking study provides detailed insights into the technical possibilities and limitations of GS and its use as a generic diagnostic workflow. We show that > 95% of known pathogenic variants, selected across the full spectrum of genetic variation, are readily detectable from GS. Modeling the impact of the transition to a generic GS strategy for our laboratory resulted in a more efficient workflow for 71% of individuals by reducing overall test complexity. A possible false negative rate of 0.3% was observed. It is possible that this potential diagnostic loss will be offset by an increase in diagnostic yield expected from GS over standard care, enabled by an evolving GS workflow, guided by better bioinformatic tools to further improve the detection of a wide variety of genomic variants and a greater understanding of non-coding and structural variant interpretation. GS thus appears a suitable generic first tier test to diagnose individuals with rare diseases.

### Supplementary Information

**Additional file 1: Table S1.** Online excel file providing overview of 1,000 individuals and workflows used. **Table S2.** Online excel file providing 1,271 genetic variants in 1,000 individuals. **Table S4.** Online excel file providing coverage statistics for 58,393 variants for which previously (likely) pathogenic variants were described. **Table S5.** Online excel file providing coverage statistics for 4,266 disease-associated genes.

**Additional file 2: Figure S1.** Scenario model to determine individuals eligible for GS-first strategy. **Figure S2.** A cohort of 1000 cases with clinically relevant variants spanning the broad range of genome diagnostics. **Figure S3.** The average output of 1000 genomes. **Figure S4.** GS Technical validation by variant type and assessment of why variants were not identified. **Figure S5.** Examples of comprehensive GS. **Figure S6.** In silico coverage statistics at variant level and disease genes. **Figure S7.** Schematic representation of referrals to Radboudumc and MUMC+ in 2022. **Figure S8.** Schematic overview of assumptions made to evaluate the impact on diagnostic yield from transition to a generic GS approach. **Table S3.** GS sensitivity: Overview of TPRs per workflow.

## Data Availability

The consent of the samples from which data was generated does not allow for broad sharing of raw FASTQ Files, and re-use of data is limited. Nonetheless, all data obtained of relevance to support the conclusions are presented in the supplementary datafiles, for which more details are available upon reasonable request from the authors.

## Abbreviations

CA: Chromosome anomaly
CNV: Copy number variants
DNA: Deoxyribonucleic acid
ES: Exome sequencing
FISH: Fluorescence in situ hybridization
FN: False negative
FNR: False negative rate
GS: Genome sequencing
MLPA: Multiplex ligation-dependent probe amplification
NGS: Next-generation sequencing
ROH: Region of homozygosity
SB: Southern blot
SMA: Spinal muscular atrophy
SNV: Single nucleotide variant
STR: Short tandem repeat
SV: Structural variant
TP: True positive
TPR: True positive rate
VCF: Variant calling format
