# Supplementary material for: Genome sequencing as a generic diagnostic strategy for rare disease
Source: Genome Med. 2024 Feb 14;16:32. doi: 10.1186/s13073-024-01301-y (PMC10868087; doi:10.1186/s13073-024-01301-y)
Supplement: Supplementary file 2 — Additional file 2: Figure S1. Scenario model to determine individuals eligible for GS-first strategy. Figure S2. A cohort of 1000 cases with clinically relevant variants spanning the broad range of genome diagnostics. Figure S3. The average output of 1000 genomes. Figure S4. GS Technical validation by variant type and assessment of why variants were not identified. Figure S5. Examples of comprehensive GS. Figure S6. In silico coverage statistics at variant level and disease genes. Figure S7. Schematic representation of referrals to Radboudumc and MUMC+ in 2022. Figure S8. Schematic overview of assumptions made to evaluate the impact on diagnostic yield from transition to a generic GS approach. Table S3. GS sensitivity: Overview of TPRs per workflow. [file 13073_2024_1301_MOESM2_ESM.pdf]

Supplement to:

Genome sequencing as a generic diagnostic strategy for rare disease

Gaby Schobers[1,2], Ronny Derks[1], Amber den Ouden[1], Hilde Swinkels[1], Jeroen van Reeuwijk[1,2], Ermanno Bosgoed[1], Dorien Lugtenberg[1], Su Ming Sun[3], Jordi Corominas Galbany[1,2], Marjan Weiss[1], Marinus J. Blok[3], Richelle A.C.M. Olde Keizer[1,2], Tom Hofste[1], Debby Hellebrekers[3], Nicole de Leeuw[1], Alexander Stegmann[3], Erik-Jan Kamsteeg[1], Aimee D.C. Paulussen[3], Marjolijn J.L. Ligtenberg[1,2], Xiangqun Zheng Bradley[4], John Peden[4], Alejandra Gutierrez[4], Adam Pullen[4], Tom Payne[4], Christian Gilissen[1,2], Arthur van den Wijngaard[3], Han G. Brunner[1,2], Marcel Nelen[1*], Helger G. Yntema[1,2*], Lisenka E.L.M. Vissers[1,2*]

[1] Department of Human Genetics, Radboudumc, Nijmegen, Netherlands

[2] Research Institute for Medical Innovation, Radboudumc, Nijmegen, Netherlands

[3] Department of Clinical Genetics, Maastricht University Medical Center, Maastricht, Netherlands

[4] Illumina Inc., Cambridge, United Kingdom

*Contributed equally

Figure S1 *Scenario model to determine individuals eligible for GS-first strategy*......2
Figure S2 *A cohort of 1000 cases with clinically relevant variants spanning the broad range of genome diagnostics*......3
Figure S3 *The average output of 1000 genomes*......4
Figure S4 *GS Technical validation by variant type and assessment of why variants were not identified*......5
Figure S5 *Examples of comprehensive GS*......6
Figure S6 *In silico coverage statistics at variant level and disease genes*......7
Figure S7 *Schematic representation of referrals to Radboudumc and MUMC+ in 2022*......8
Figure S8 *Schematic overview of assumptions made to evaluate the impact on diagnostic yield from transition to a generic GS approach*......9
Table S3 *GS sensitivity: Overview of TPRs per workflow*......10

(Additional file 1)
Table S1 *Online excel file providing overview of 1,000 individuals and workflows used*
Table S2 *Online excel file providing 1,271 genetic variants in 1,000 individuals*
Table S4 *Online excel file providing coverage statistics for 58,393 variants for which previously (likely) pathogenic variants were described.*
Table S5 *Online excel file providing coverage statistics for 4,266 disease-associated genes*

Figure S1: Scenario model to determine individuals eligible for GS-first strategy

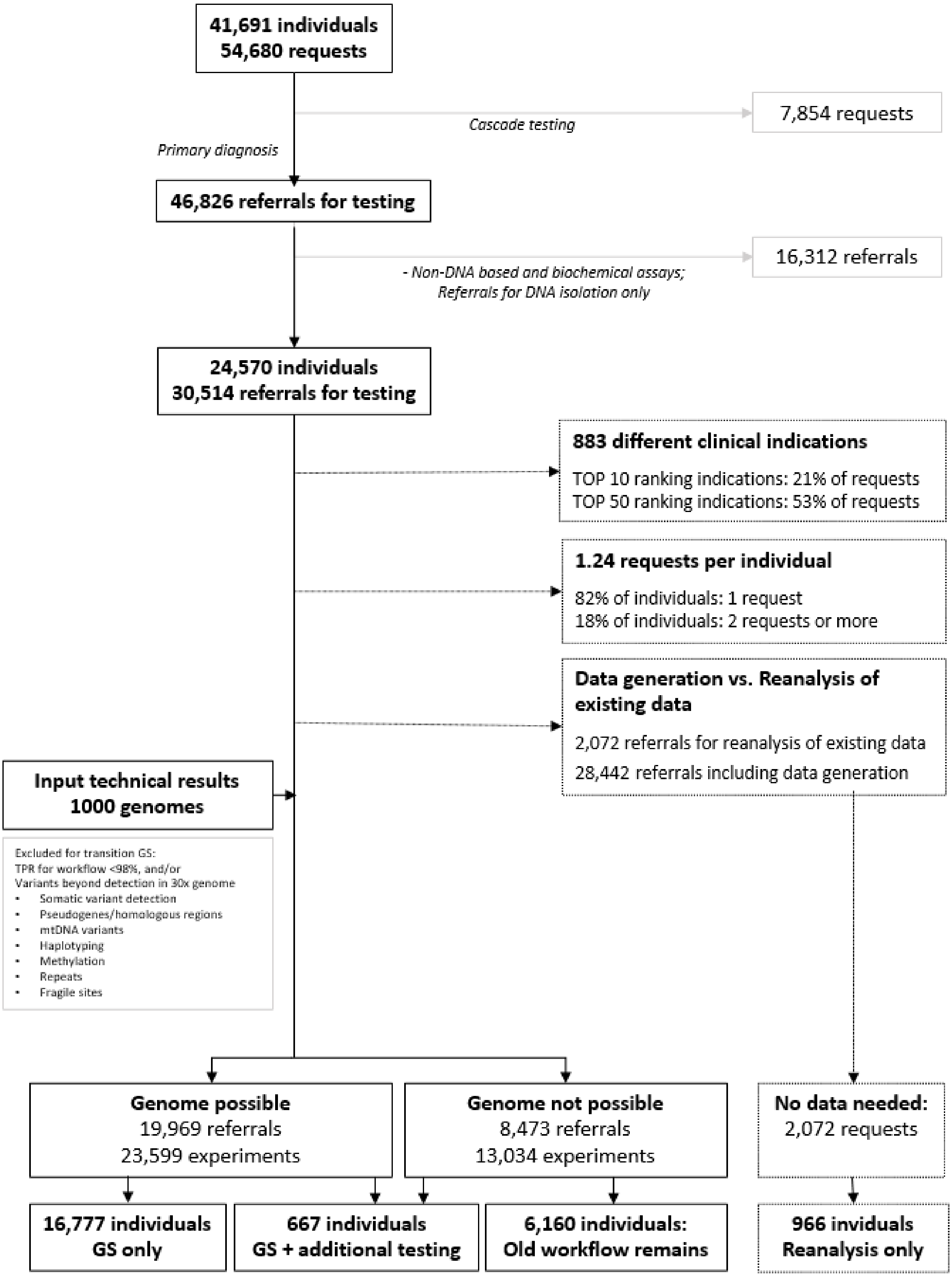

Figure S2: A cohort of 1000 cases with clinically relevant variants spanning the broad range of genome diagnostics.

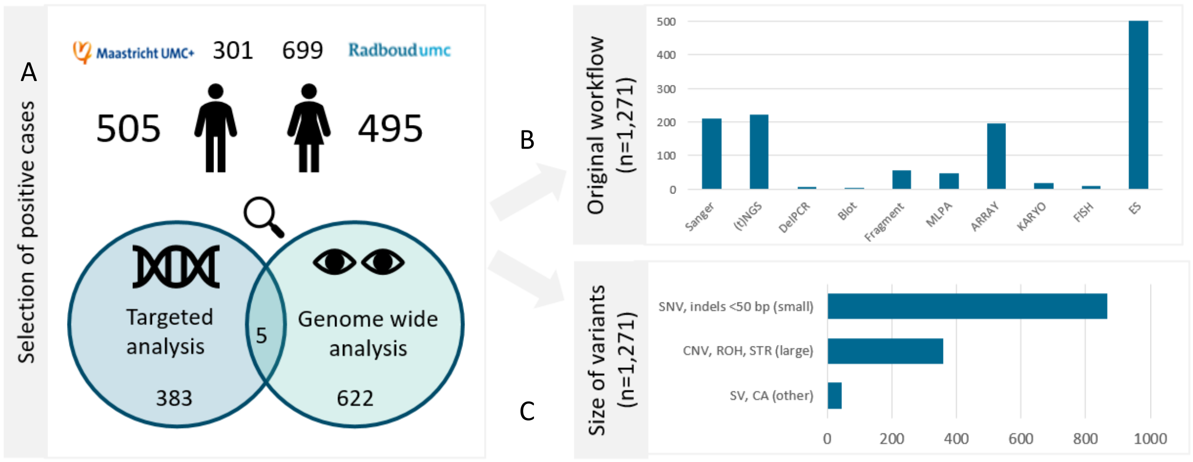

A The 1000 genomes cohort consisted of 505 males and 495 females, who were genetically diagnosed in the Radboudumc or Maastricht UMC+ in 2018. The assays that were performed to find these diagnoses were either targeting specific variants and single (or a small set of) genes or complete gene panels or chromosomes were analyzed based on the patient's phenotype. **B** In these cases, a total of 1,271 variants were identified, requiring >10 different workflows to diagnose them. **C** The variants were grouped in small (<50 bp), large (50 bp and up), and other variants (SVs and CA).

Abbreviations: targeted next generation sequencing ((t)NGS), deletion polymerase chain reaction (DelPCR), multiplex ligation-dependent probe amplification (MLPA), fluorescent in situ hybridisation (FISH), exome sequencing (ES), single nucleotide variants (SNV), short tandem repeat expansions (STRs), regions of homozygosity (ROH), copy number variants (CNV), structural variants (SV), chromosome anomalies (CA)

Figure S3: The average output of 1000 genomes.

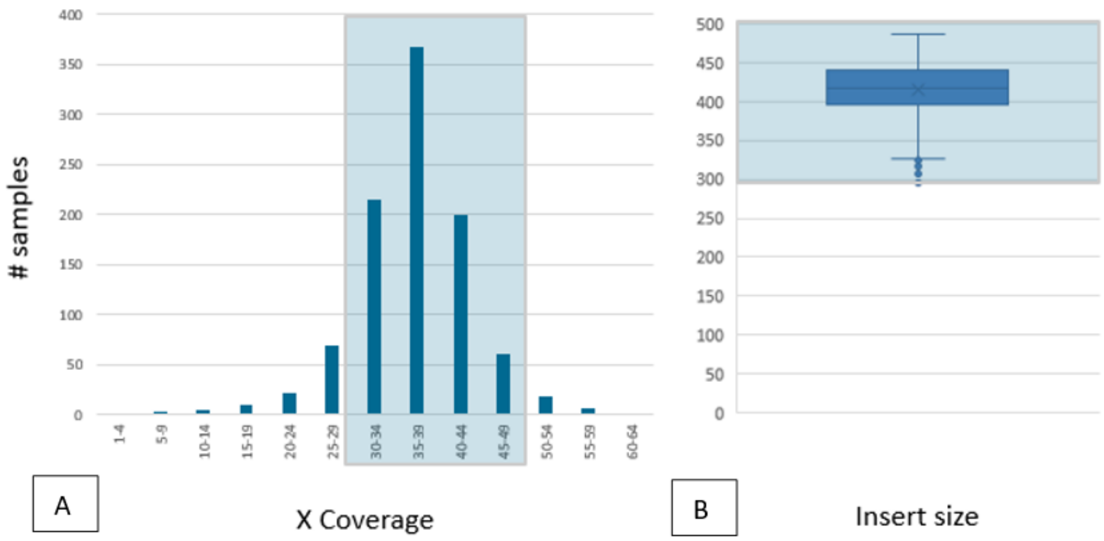

A As multiple observations per base are needed to come to a reliable base call, the recommended sequencing depth for genome sequencing is 30x to 50x. **B** Insert sizes are also important for the sequencing. For efficient sequencing, small insert sizes (risk of overlapping paired sequences) as well as larger fragments (decrease of cluster efficiency) must be avoided. We therefore aimed for a 300-500bp range for our 2x150bp paired-end sequencing. In this project we reached an average sequencing depth of 37x and an insert size of around 400-450bp.

Figure S4: GS Technical validation by variant type and assessment of why variants were not identified

A

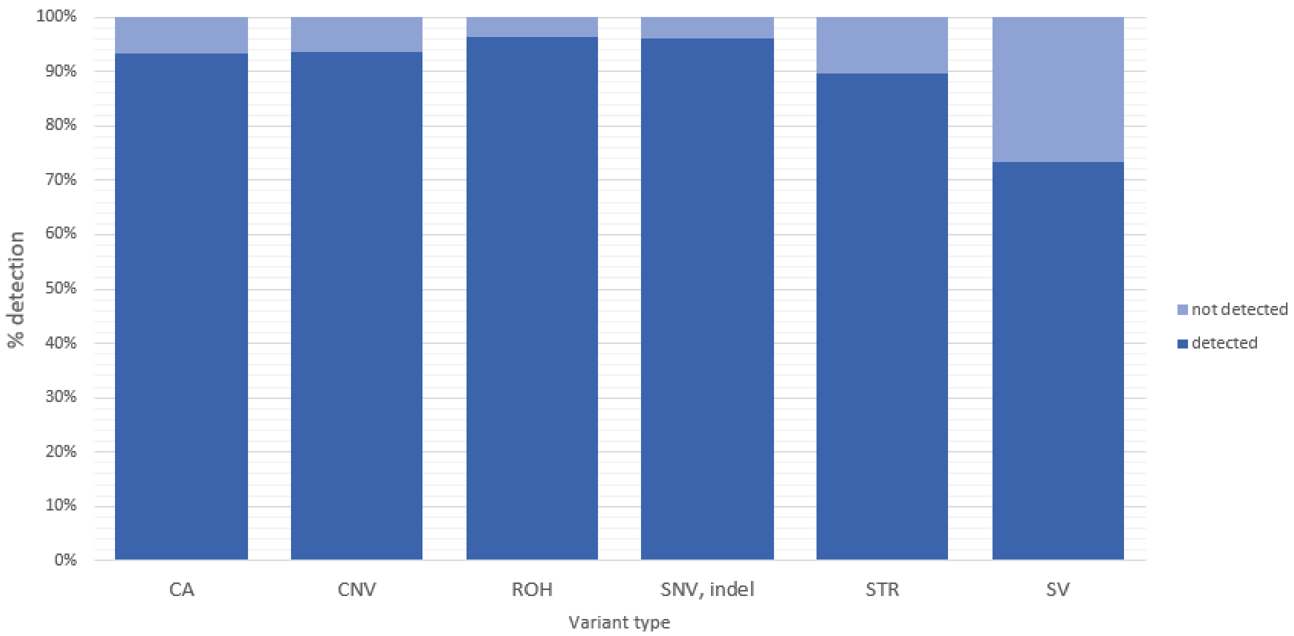

B

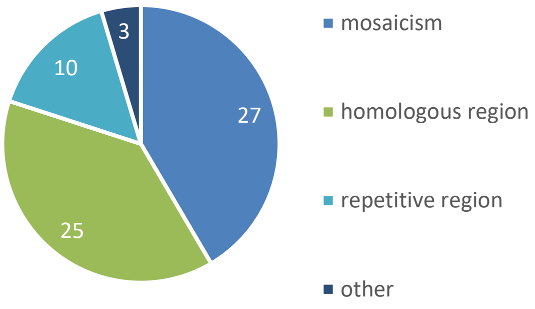

C

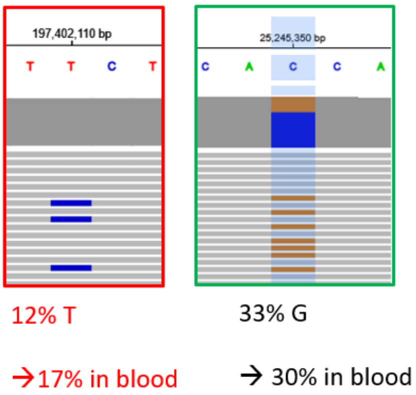

A In total, 94.9 % (1,206/1,271) of all variants were detected with GS. Small variants (<50bp) were detected in 96.1% (833/867), large variants (123 bp - 72.8Mb) in 93.3% (334/359), and other variants in 86.7% (39/45). The total list of variants and whether they were present in the GS data ('detected' vs. 'not detected') can be found in **Supplementary Table S2**. **B** In the 5% undetected variants (N=65), we identified common themes that are attributable to short-read 30x GS and downstream analysis. Undetected variants were mostly found in mosaic cases (n=27, 2.4-20%), homologous regions (n=25), i.e. pseudogenes or paralogues genes, or likewise in repetitive regions (n=10), i.e. repeats, telomeres or centromeres, and 3 others. **C** A mosaic variants in the *SF3B1* gene (Chr2(GRCh38):g.197402110T>C), which was originally detected with a targeted NGS approach in 17% of the blood sample, was present in 6/50 (12%) of the reads and not present in the VCF file of the GS data. A mosaic variant in the *KRAS* gene (Chr12(GRCh38):g.25245350C>G, originally detected with a targeted NGS approach in 30% of the blood sample, was present in 15/46 (33%) of the reads and in the VCF file of the GS data.

Figure S5: Examples of comprehensive GS

A

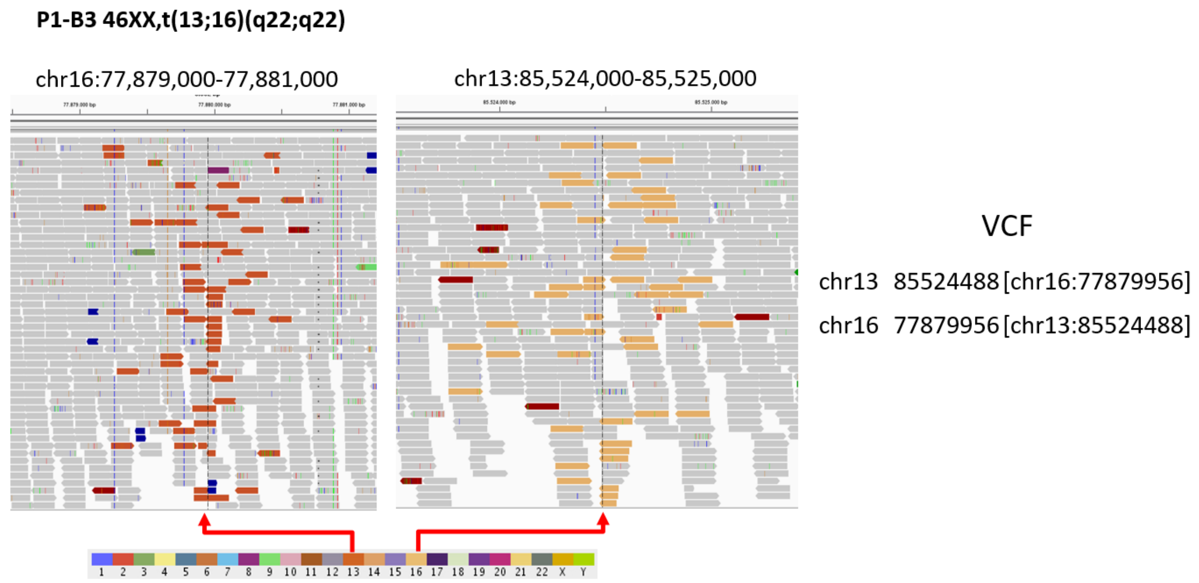

B

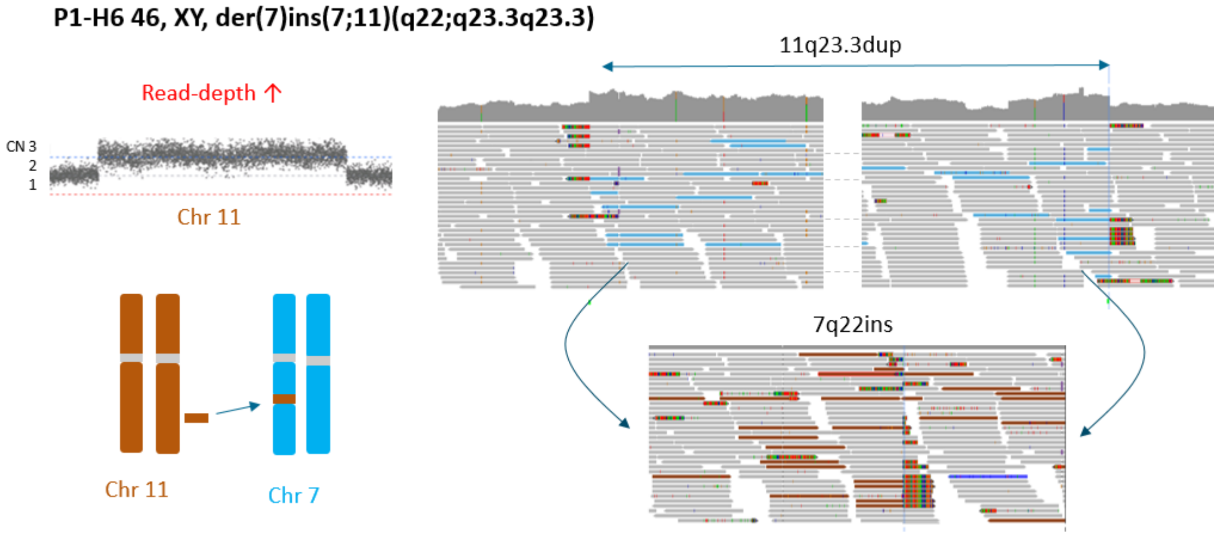

C

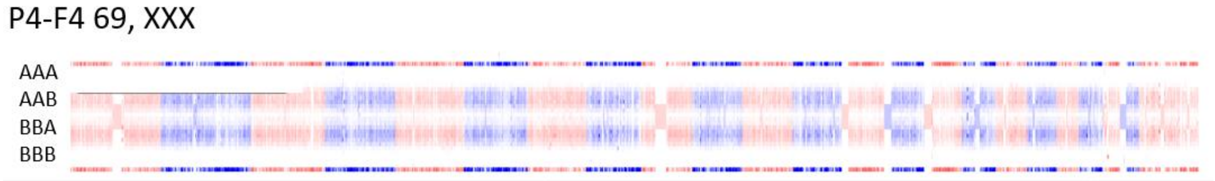

A Based on visual inspection and targeted manual search of the variant calling format (VCF) file, we could identify the previously detected translocation between chromosomes 13 and 16. **B** Likewise, we detected a copy-number gain on chromosome 11, which translocated to chromosome 7. In the diagnostic trajectory this derivative chromosome was detected with a targeted FISH analysis performed subsequent to an array analysis, in which only the gain was identified. **C** GS B-allele frequency plots can identify triploidies.

Figure S6: *In silico* coverage statistics at variant level and disease genes

A:Coverage statistics for 794 detected SNVs from the 1000 Genomes

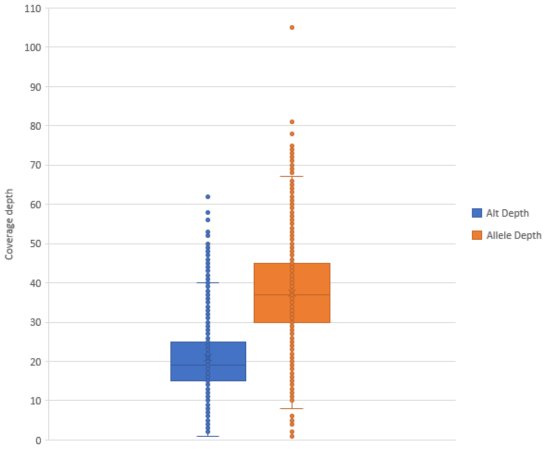

B: Coverage statistics of 58,393 ClinVar and VKGL variants

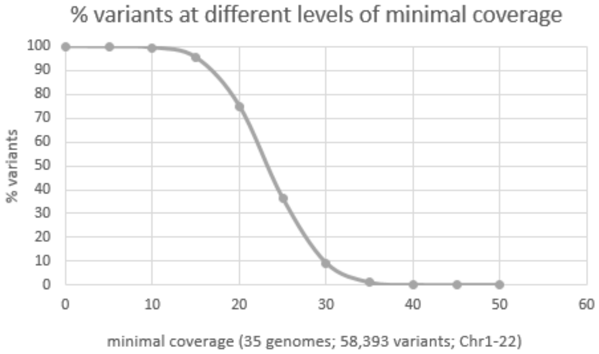

C-D: Coverage statistics of 4,266 disease genes

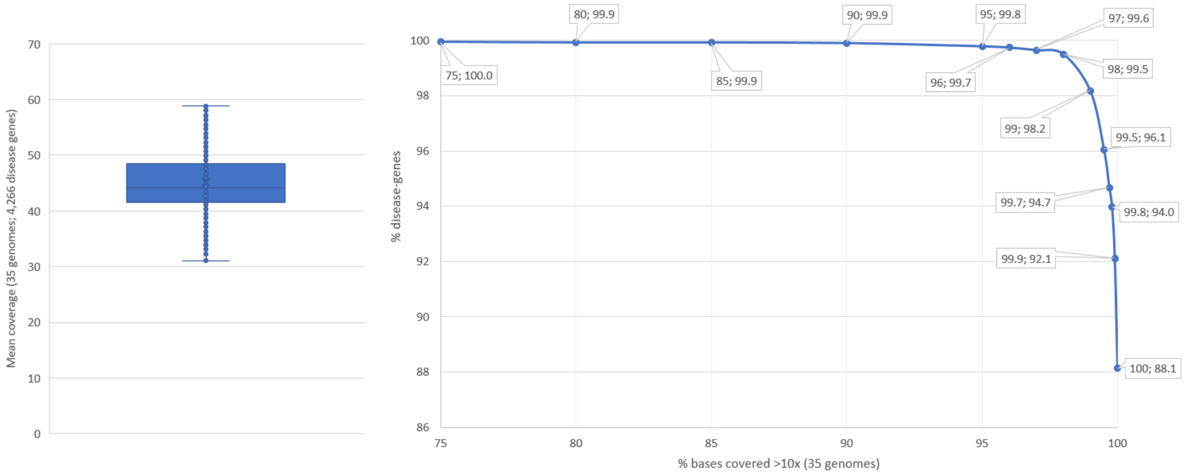

A Coverage data of 794 detected SNVs in our cohort, where allele depth ranged from 1-105, and (variant) alternative allele depth ranged from 1-62, with a 13-100% variant range. **B** Sequence depth at genomic positions that are known to harbor (likely) pathogenic variation and **C** Mean coverages for all coding positions of genes with well-established rare disease associations were calculated from 35 randomly selected genomes. **D** The fraction of genes versus the percentage of bases of the gene with ≥10x coverage.

Figure S7: Schematic representation of referrals to Radboudumc and MUMC+ in 2022

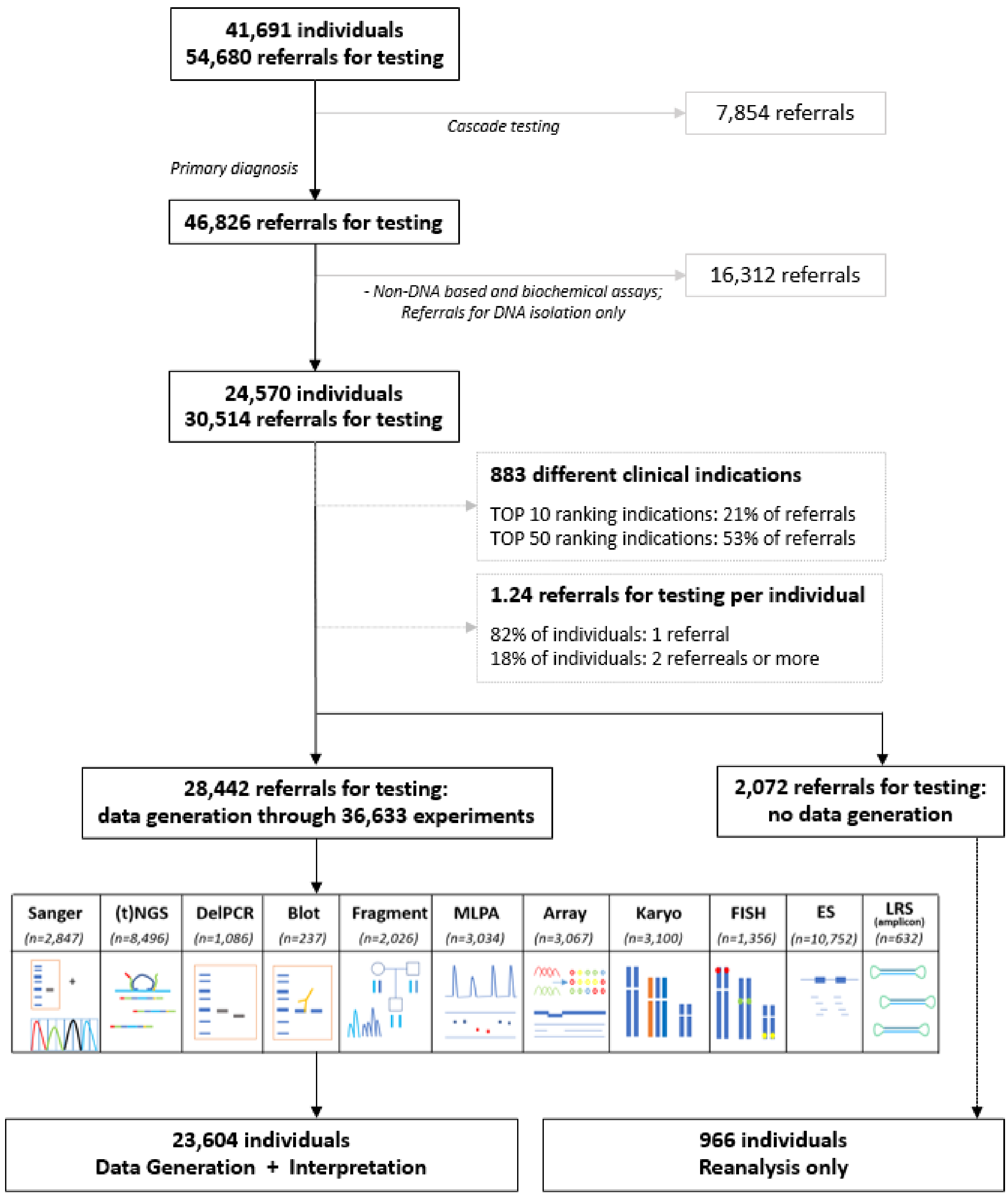

Figure S8: Schematic overview of assumptions made to evaluate the impact on diagnostic yield from transition to a generic GS approach

A

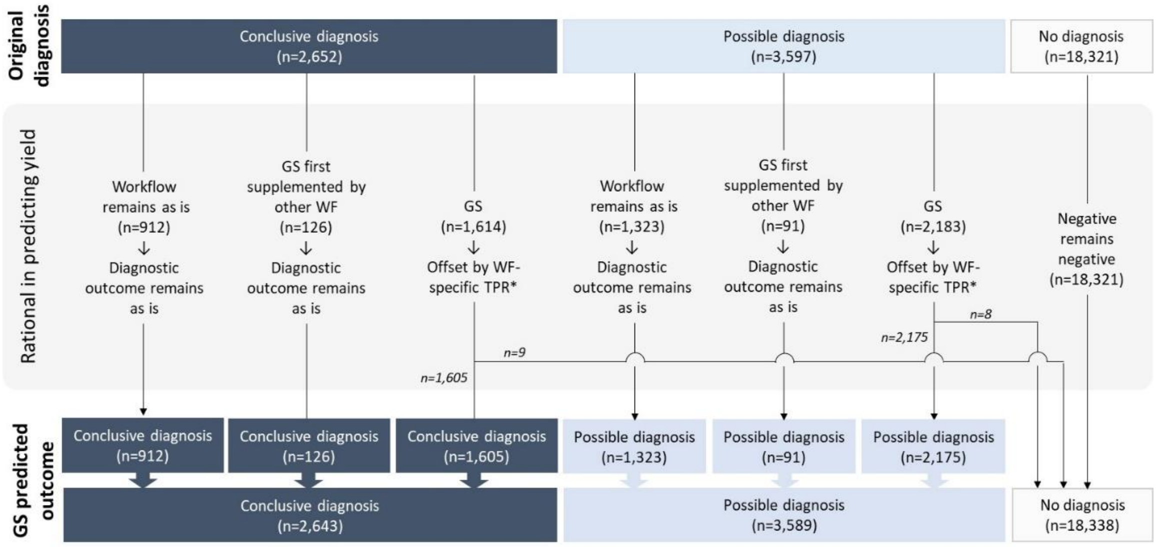

B

Workflow leading to (possible) diagnosis	Conclusive	Possible	TPR	Conclusive	Possible	Delta
(t)NGS	197	662	100,00%	197	662	-
ES	1253	1260	99,40%	1245	1253	15
Sanger	74	176	100,00%	74	176	-
Array	16	17	98,80%	15	16	2
Fragment	61	7	100,00%	61	7	-
MLPA	4	2	100,00%	4	2	-
DelPCR	9	24	100,00%	9	24	-
Blot	0	35	100,00%	0	35	-

A) Based on clinical referrals being transferable to generic GS, the impact on diagnosis was evaluated for all 24,570. Top row shows original diagnosis per individual, where '*n*' refers to number of individuals; *Offset with workflow specific TPRs are provided in **B**. Assuming all negative diagnoses remain negative, this translates to a possible false negative diagnostic rate of 0.3% (17/6232).

Table S3: GS sensitivity

*Excluded indications: Adenomatous polyposis coli, Chronic lymphocytic leukemia, PTEN Hamartoma tumor syndrome (diagnostic referrals that are under suspicion of harboring mosaic variants and/or added to include mosaic variants although not primarily aimed at germline testing); Excluded variants: mosaic variants <20%, variants in the *CYP21A2*, *SMN1*, *OTOA*, *STRC* or OPSIN genes.

TPR≥98% indicated by grey marking

# variants<br>Workflow	1) Technical validation				2) Technical validation + exclusion expected false negatives*			
	positive	false negative	total	TPR	positive	false negative	total	TPR
Sanger	197	12	209	94.3%	178	0	178	100.0%
(t)NGS	209	13	222	94.1%	193	0	193	100.0%
DelPCR	6	1	7	85.7%	4	0	4	100.0%
Blot	2	0	2	100.0%	2	0	2	100.0%
Fragment	51	6	57	89.5%	51	6	57	89.5%
MLPA	44	4	48	91.7%	33	0	33	100.0%
Array	183	14	197	92.9%	168	2	170	98.8%
Karyo	15	4	19	78.9%	15	1	16	93.8%
FISH	8	2	10	80.0%	8	2	10	80.0%
ES	491	9	500	98.2%	486	3	489	99.4%
**Total**	**1206**	**65**	**1271**	**94.9%**	**1138**	**14**	**1152**	**98.8%**
**Type variant**								
SNV, indels	827	34	861	96.1%	789	3	792	99.6%
STR	52	6	58	89.7%	52	6	58	89.7%
ROH	26	1	27	96.3%	24	0	24	100.0%
CNV	262	18	280	93.6%	239	2	241	99.2%
CA	28	2	30	93.3%	23	0	23	100.0%
SV	11	4	15	73.3%	11	3	14	78.6%
**Total**	**1206**	**65**	**1271**		**1138**	**14**	**1152**	

Abbreviations: targeted next generation sequencing ((t)NGS), deletion polymerase chain reaction (DelPCR) multiplex ligation-dependent probe amplification (MLPA), fluorescence in situ hybridisation (FISH), exome sequencing (ES), single nucleotide variants (SNV), short tandem repeat expansions (STRs), regions of homozygosity (ROH), copy number variants (CNV),), chromosome anomalies (CA), structural variants (SV)
